# Rectal prolapse: mechanical resection sec. altemeier (Reverse Transtar)

**DOI:** 10.1186/1471-2318-11-S1-A2

**Published:** 2011-08-24

**Authors:** D Barbato, P Sorrentino, R Vincenti

**Affiliations:** 1 UOC di Chirurgia Generale - Ospedale Fatebenefratelli Napoli, Italy

## Background

The recent literature confirms that perineal rectosigmoidectomy sec. Altemeier in the case of a full-thickness rectal prolapse represents, to the state, the procedure of choice even if burdened by a relatively high recurrence rate. Some authors oppose the transabdominal rectopexy to the perineal resection with or without resection of the sigma, with or without the aid of prosthesis, sustaining the Altemeier cause of fecal incontinence.

## Materials and methods

Our experience includes eight women with a mean age of 74 aa, with full-thickness rectal prolapse from 8 cm to around 16 cm, all burdened by incontinence to liquid stools, in absence of systemic or associated organ pathologies.

Patients are submitted to colonoscopy, anorectal manometry and 3D-endoanal ultrasonography.

The colonoscopy was, in all the examined cases, negative for a greater pathology.

Regarding the manometry the preoperative remaining pressure of anal canal (RP) was close to zero with a total absence of rectoanal inhibitory reflex (RAIR). In the manometry after PNE the average of the remaining pressure was 45 cm H2O with recurrence of rectoanal inhibitory reflex (RAIR).

The ultrasound in all the cases showed the thinning of the sphincterial apparatus.

All patients underwent (for 30 days) temporary percutaneous nerve evaluation (PNE). During this period all the patients solved the external prolapse, also recovering continence of gas but reporting the onset of obstructed defecation. Between 1 and 2 months after the end of the neuromodulation the patients were operated on.

The technique of choice was a purely mechanical resection sec. Altemeier, through the use of a stapler Contour Transtar (with the help of 5-7 refills) and GIA. The resection was deliberately set at about 2 cm from the anal-rectal junction because we think that leaving only all the perceptive mucosa the patients may continue to have control of defecation.

## Results

With regard to the state of the patient, after a median follow-up of 1 year (2 months-1.2 years) no patient had recurrent prolapses but, above all, reported total anal continence. There were no postoperative complications such as postoperative hemorrhages, abdominal infections, or anastomotic leakages.

## Conclusions

The mechanical resection sec. Altemeier (Reverse Transtar) presents a low risk of mortality and immediate complications, even in elderly patients. Functional results and a long-term recurrence rate must be investigated further.

